# Patterns and predictors of clinician use of interoperability tools

**DOI:** 10.1093/jamiaopen/ooag116

**Published:** 2026-06-11

**Authors:** Sadaf Ashtari, A Jay Holmgren, Hector P Rodriguez

**Affiliations:** Information Systems & Business Analytics, California State University, Sacramento, CA 95819, United States; Center for Health Management & Policy Research, School of Public Health, University of California, Berkeley, CA 94720, United States; Division of Clinical Informatics and Digital Transformation, Department of Medicine, University of California, San Francisco, CA 94143, United States; Center for Health Management & Policy Research, School of Public Health, University of California, Berkeley, CA 94720, United States; Division of Health Policy & Management, University of California, Berkeley, CA 94720, United States

**Keywords:** interoperability, electronic health records, care everywhere, clinician behavior, health information exchange

## Abstract

**Objectives:**

Clinicians’ adoption of interoperability tools influences care quality, but evidence of actual use is limited. We analyze clinicians’ use of outside records delivered via Epic Care Everywhere (CE), focusing on use frequency and predictors such as gender, experience, specialty, and role. Differences between pre-pandemic (2018-2019) and pandemic (2020-2021) periods are also examined to see how COVID-19 affected use of outside records.

**Materials and methods:**

De-identified EHR metadata from UCSF clinicians (*n* = 1442) during pre-pandemic and pandemic periods, totaling 686 797 clinician-day observations, were analyzed. We measured usage intensity (mean CE lookups per appointment) and breadth (percentage of appointments with ≥1 lookup). Generalized linear models (GLMs) with a negative binomial distribution for overdispersion were used to estimate predictors of intensity.

**Results:**

CE usage intensity rose by 43.0% post-COVID-19 onset compared with the pre-pandemic. Clinician specialty most strongly predicted use, with Nephrology and Cardiology showing the highest breadth (56.0% and 53.0% of visits, respectively), while Dermatology (13.2%) and Pediatrics (23.4%) were lowest. Residents used CE at 23.0% greater intensity than attendings, and each additional year of experience was linked to a 0.57% decrease in intensity.

**Discussion:**

Clinician use of interoperability tools was higher in specialties such as Nephrology and Cardiology that require more care coordination, and among less experienced clinicians including resident physicians. Use increased after the pandemic began, likely due to ongoing adoption trends and increased clinical demands during system strain and uncertainty.

**Conclusion:**

These findings underscore the critical importance of considering clinician behavior and contextual factors, such as specialty care needs, in addition to technical capabilities, when promoting the adoption and use of interoperability tools.

## Background and significance

Electronic Health Records (EHRs) have transformed healthcare delivery in many ways by digitizing patient information and improving data accessibility^1^; however, many of the theorized benefits of EHRs are contingent on seamless interoperability^2^—the ability of 2 or more systems to exchange health information and meaningfully use it once received.^3^ Efficient, standardized data exchange between healthcare providers and various organizations is crucial, and it can enhance patient outcomes, coordinate care, and improve population health management. A fully interoperable EHR system supports clinical workflow efficiency, increases patient safety, reduces redundant testing, and strengthens public health surveillance.^4–6^ Interoperability has been linked to lower mortality rates among vulnerable populations, such as elderly patients with Alzheimer’s disease,^7^ and has improved emergency department performance and health system responsiveness to public health crises.^5^

In the early 2010s, health information exchange in the United States was limited, and fewer than 25% of hospitals could fully share and integrate external patient information.^8^ Most health systems relied on outdated methods like fax or phone for data sharing. By 2021, interoperability had significantly improved, with 62% of hospitals engaging in all 4 data exchange—sending, receiving, finding, and integrating information—and 74% able to integrate outside data into their EHRs.^8^ Overall, U.S. healthcare has shifted from siloed systems to broader, albeit still incomplete, data-sharing capabilities. Despite this increased connectivity, many of the benefits of interoperability have yet to be realized, including a lack of reductions in duplicative care.^9^ It may be that while healthcare delivery organizations are increasingly connected, front-line clinicians are not accessing outside records or changing their behavior in response to this newly available data. In this study, the term interoperability tools refers to EHR-integrated health information exchange (HIE) functionality, specifically Epic’s Care Everywhere, that allows clinicians to find, retrieve, and view outside records within their workflow. Interoperability encompasses a broader range of definitions and technical standards, from syntactic data exchange to full semantic integration; while this study’s operationalization does not capture all of these dimensions, it reflects a meaningful and measurable form of clinician-facing data exchange.

Care Everywhere (CE) is a HIE platform developed by EHR vendor Epic Systems, that facilitates the secure and standardized exchange of patient information between different healthcare organizations using Epic’s EHR, as well as non-Epic sites participating in certain national HIE networks. It allows authorized clinicians to access patient records from other participating healthcare facilities during treatment, enhancing care coordination and potentially reducing redundant testing and procedures.

Despite national efforts to build technical and regulatory infrastructure for interoperability, there is limited research on how clinicians actually use these tools in practice. This includes who uses them, how often, and why.

While prior studies^10–12^ have documented clinician interaction with HIE systems, the landscape of interoperability has advanced with the integration of vendor-based exchange tools and the rapid digital adoption during the COVID-19 era. Updating this evidence with post-pandemic data offers new insight into current clinician-level use patterns. Earlier studies have also examined HIE trends and integration within EHRs,^13^^,^^14^ underscoring the need for continued monitoring of clinician-level use. Most existing research has focused on organizational participation in HIE, with studies showing that by 2019, about 65% of physicians were involved in some form of HIE.^15^^,^^16^ This involvement was particularly high among primary care physicians,^17^^,^^18^ those in large practices,^18^ participants in value-based payment models such as accountable care organizations (ACOs), and large integrated delivery networks (IDNs).^19^ However, this system-level perspective provides limited insight into the actual use of interoperable data and individual clinician use patterns. Understanding clinician-level factors is crucial for addressing the remaining gaps in interoperability adoption, specifically the “last mile” of encouraging the actual use of outside records to reduce duplicative services and improve care quality and coordination. To our knowledge, no empirical study has quantified clinician‑level use of Epic’s Care Everywhere interoperability platform.

## Objective

The primary goal of this study is to examine clinicians’ use of outside records delivered via a vendor-based interoperability tool, specifically Epic’s Care Everywhere, at UCSF Health, a large academic medical center where Care Everywhere is the dominant interoperability channel.^20^ This study aims to analyze the frequency and depth of clinicians’ utilization of external patient information, specifically assessing the intensity (mean lookups per visit) and breadth (percentage of visits with any lookup) of Care Everywhere use. Additionally, this study identifies predictors of clinician use by evaluating factors such as clinician gender, years of professional experience, medical specialty, and compares data usage between the pre-pandemic (2018-2019) and pandemic (2020-2021) periods.

## Materials and methods

### Data and sample

This quantitative retrospective observational study involves the secondary analysis of existing, de-identified electronic health record (EHR) data. For ethical considerations, this study was deemed exempt by the UCSF Institutional Review Board (IRB) approval #21-34512. The study population included data extracted from the UCSF Health Epic Clarity database for 2 distinct periods: 2018-2019 (pre-pandemic) and 2020-2021 (during the COVID-19 pandemic). This study is limited to a single institution, UCSF Health, whose Care Everywhere implementation connects all major community and referral partners on Epic. This design improves internal validity because it eliminates cross-system differences in data completeness or network participation. Records with missing key variables (eg, gender and number of appointments scheduled) were excluded from relevant analyses (*n* = 22). The final analytic sample includes 1442 individual clinicians.

### Measures

The dependent variables in this study were the total number of patients for whom the clinician accessed external information (Care Everywhere) on a given day and the total number of scheduled patients for whom external data was accessed on that same day. These variables reflect both overall CE use, which may include access to outside records for care management or when prompted by asynchronous communication such as a patient portal message, as well as targeted CE use for a specific encounter. Two metrics were defined to distinguish how often versus how deeply clinicians consult external records: intensity, the total number of CE lookups divided by the total number of scheduled visits (mean lookups per visit), and breadth, the percentage of visits in which at least 1 lookup occurred. Intensity reflects lookup volume relative to visit count, while breadth indicates the share of unique patient encounters that engaged CE at all. In most specialties, breadth exceeds or approximates intensity, since a single visit may prompt multiple lookups, but intensity cannot exceed breadth when only 1 lookup occurs per visit. The constructs of breadth and intensity are grounded in the literature on information systems, which distinguishes between the extent and depth of technology use.^21–24^ The operationalization used in this study aligns with these established definitions, adapted to clinician-level interoperability tool use.

In addition, day-level use was calculated as the proportion of active clinic days (days with ≥ 1 scheduled appointment) on which at least 1 CE lookup occurred, providing a daily view of clinicians’ reliance on external records. Our clinician-day observations reflect a 3:00 am to 3:00 Pm day definition to capture all activity relevant to the workday. As the clinicians are based at an academic medical center with research, teaching, and service responsibilities, we differentiate between active and non-active days. This distinction matters because clinicians’ EHR activity is not limited to direct patient visits. On non-active clinic days (zero scheduled appointments), clinicians often do follow-up documentation, reply to messages on patient portals, review test results, or prepare for future visits. Including these “non-active” days helps us better capture the complete extent of clinician interaction with the EHR and other data sources, rather than focusing only on in-clinic activities.

The independent variables included the gender of the clinician, specialty (internal medicine, pediatrics, etc), role (attending physician, resident, or advanced practice clinician (APC)), years of professional experience, calculated as the number of years from medical school graduation to the date of observation, which was treated as a continuous variable in regression models; categorical groupings (eg, 0-5 and 6-10 years) were used only for descriptive summaries, and time period (2018-2019 vs 2020-2021), representing pre- and post-COVID eras. Prior studies have reported gender differences in EHR use and EHR-related workload, including time spent in the EHR, after-hours EHR time, inbox work, and documentation patterns.^25–29^ Therefore, clinician gender was included as a covariate to adjust for potential differences in baseline EHR use behaviors that could confound interoperability tool use.

### Analysis

Descriptive statistics were used to characterize CE usage by clinician characteristics and to summarize key metrics such as mean CE usage intensity per appointment and the proportion of active clinic days involving CE adoption.

This study examined the adjusted associations between clinician characteristics and Care Everywhere (CE) usage using a series of regression models. Negative Binomial (NB) and Poisson regression models were separately estimated to model CE usage as a count-based outcome, adjusting for clinician gender, role (attending, resident, APC), specialty, years of experience, and period (2018-2019 vs 2020-2021.

The dependent variable, the number of scheduled patients for whom a clinician accessed external records via Care Everywhere on a given day, is count measure. For visit-level behavior, the number of appointments per day was included as a log-offset term in count-based models to normalize Care Everywhere (CE) lookups by visit volume, a standard approach in rate modeling. The number of scheduled appointments per day was included as an offset to model CE usage per scheduled appointment.

Generalized estimating equations (GEEs) were used to account for the repeated measures in our dataset as each clinician contributes multiple daily observations, which are likely correlated. Because clinicians within the same specialty may have correlated use patterns, within-clinician correlation was accounted for using GEE with robust standard errors, which produce consistent estimates even in the presence of clustering at higher levels (eg, specialty). A working correlation structure was specified, and robust (sandwich) standard errors were used to obtain population‐averaged estimates of the associations of gender, specialty, role, experience, and time period on CE usage per appointment, adjusting for the number of appointments per day. This approach ensures our measures are not biased by serial autocorrelation as observations from the same clinician are not independent and confirms that the period effect and other key predictors remain robust at the population level. GEE was selected as our main method because it measures population-average (marginal) effects that match our descriptive goal and offer reliable inference even if the within-clinician correlation is misspecified. In contrast, fixed-effects models are better suited for isolating within-clinician causal effects and do not provide coefficients for time-invariant clinician traits unless these traits are included as interactions.

Results are reported as incidence rate ratios (IRRs) with 95% confidence intervals; 1-sided *P* value<.05 were considered statistically significant. Analyses were conducted in R 4.1.2 (RStudio 1.4.1106) using the packages dplyr, geepack, MASS, and broom.

## Result

### Sample characteristics

The dataset comprised 686 797 clinician-day records for 2 time periods: September 2018-September 2019 (pre-COVID) and September 2020-September 2021 (during COVID). Of the 1442 clinicians, 779 appeared in both periods. Out of all clinician-day records, only 22.7% (156 040 days) were considered active, which means the clinician had at least 1 scheduled appointment. The remaining 77.3% (530 756 days) were non-active, with no appointments scheduled but at least some EHR activity. The clinician population is 64.0% female (*n* = 956) and 36.0% male (*n* = 538). In terms of clinical occupation, physicians made up the largest group (*n* = 744, 51.6%), followed by residents (*n* = 577, 40.0%) and advanced practice clinicians (APCs, *n* = 174, 12.1%).

Internal Medicine was the most common specialty (*n* = 368, 25.5%), followed by Neurology (290, 20.1%) and OB/GYN (181, 12.5%). General Surgery (42, 2.9%) and Family Medicine (29, 2.0%) had the fewest clinicians.

The number of scheduled appointments per day was consistent throughout weekdays, averaging 8.6 appointments on Mondays and 8.0 on Fridays. Both Saturdays and Sundays experienced the lowest volumes of appointments. Key counts and percentages are summarized in Table 1; a full specialty breakdown and the detailed weekday appointment distribution are provided in Tables S1 and S2.

**Table 1.** Sample characteristics.

Characteristic	*n*	%
**Clinicians (unique)**	**1442**	
** Female**	956	64.0
** Male**	538	36.0
**Clinical role**		
** Physician**	744	51.6
** Resident**	577	40.0
** Advanced practice clinician (APC)**	174	12.1
**Top specialties^a^**		
** Internal Medicine**	368	25.5
** Neurology**	290	20.1
** OB/GYN**	181	12.5
** All other specialties**	603	41.9
**Clinicians present in both periods**	**779**	54.0
**Clinician-day records**	**686 797**	
** Pre-COVID (Sep 2018-Sep 2019)**	336 103	48.9
** COVID (Sep 2020-Sep 2021)**	350 694	51.1
** Active days (≥ 1 scheduled visit)**	156 040	22.7
** Non-active days (0 scheduled visits)**	530 756	77.3
**Mean appointments per active day**	8.46 ± 3.05^b^	

a Remaining specialties (dermatology, pediatrics, cardiology, etc) are grouped under “All other specialties” for brevity; full breakdown in Table S1.

b Pooled mean ± SD across all active clinician-days.

### Descriptive analysis of care everywhere usage

The intensity of CE use was almost similar for male and female clinicians. Female clinicians (*n* = 921) conducted 0.456 lookup per appointment, while male clinicians (*n* = 520) performed 0.453 CE lookup per appointment. Although gender differences in CE usage intensity were minimal, distinctions were more significant across clinical roles. Residents demonstrated the highest intensity averaging 0.49 lookup per visit. Table 2 illustrates the usage of Care Everywhere (CE) by role.

**Table 2.** Care everywhere (CE) usage intensity and appointment volume by clinical role (mean ± SD).

Clinical role	Number of clinicians	CE per Appt (mean ± SD)	Mean scheduled appointments per active day (± SD)
**Resident**	577	0.49 ± 0.32	5.36 ± 2.94
**Advanced practice clinician (APC)**	174	0.48 ± 0.30	7.34 ± 5.6
**Attending physician**	744	0.44 ± 0.33	9.14 ± 7.34

Clinicians with less experience used CE more intensively. Mean lookups per appointment dropped from 0.499 for the least experienced clinicians (0-5 years) to 0.409 for those with 35-40 years of experience. This descriptive trend is consistent with the adjusted regression results, which showed that experience, modeled as a continuous variable, was associated with a 0.6% decrease in usage per year (IRR = 0.99, *P* < .001).

Substantial differences in CE usage were observed across specialties. Among the 11 specialties recorded in the dataset, Nephrology (intensity = 0.561 lookups/visit; breadth = 56.0% of visits with ≥1 lookup) and Cardiology (0.553; 53.0%) had the highest use, whereas Dermatology (0.187; 13.2%) and Pediatrics (0.259; 23.4%) recorded the lowest. Day-level use, defined as the percentage of clinic days with any CE adoption, followed a similar pattern (eg, 83.5% for Nephrology vs 73.8% for Dermatology). The CE usage by specialty is shown in Table 3.

**Table 3.** Care everywhere (CE) usage by specialty: intensity (mean ± SD), breadth, and day-level use.

Specialty	Intensity (mean CE per Appt ± SD)	Breadth (% visits with lookup)	Day-level use (% active days)
**Nephrology**	0.561 ± 0.34	56.0	83.5
**Cardiology**	0.553 ± 0.36	53.0	80.9
**Internal medicine**	0.532 ± 0.27	51.5	91.8
**Hematology/oncology**	0.524 ± 0.33	51.4	84.5
**Family medicine**	0.496 ± 0.25	48.1	94.5
**Neurology**	0.467 ± 0.34	43.8	79.3
**Obstetrics and gynecology**	0.455 ± 0.31	39.4	85.4
**General surgery**	0.370 ± 0.35	39.0	65.1
**Otolaryngology**	0.343 ± 0.28	32.3	78.5
**Pediatrics**	0.259 ± 0.24	23.4	75.7
**Dermatology**	0.187 ± 0.23	13.2	73.8

Temporal comparisons indicated that clinicians increased CE usage during the COVID-19 period (0.557) compared to the pre-pandemic period (0.414), even with fewer scheduled appointments per day.

### Adjusted analyses

Comparing models suggested that the NB rate model has a substantially lower Akaike Information Criterion (AIC = 598, 034) than the Poisson rate model (AIC = 602, 761), indicating a better model fit. Additionally, McFadden’ s pseudo-*R*^2^ was 0.18, indicating a substantial improvement in model fit compared with the null model. Consequently, NB rate models were adopted as the main regression models.

**Figure 1.**
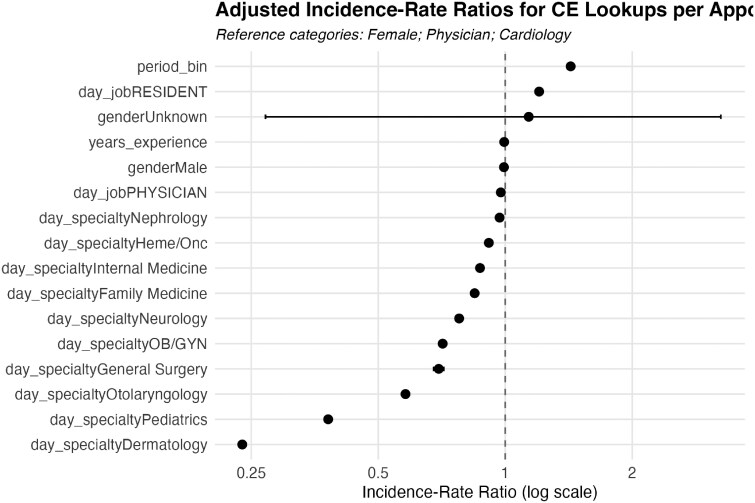
Adjusted IRRs for CE lookup per appointment. Reference categories are Female (gender), Clinician (role), and Cardiology (specialty). Period is coded as 2020-2021 vs 2018-2019. Estimates come from a negative‐binomial rate model offset by log (appointments per day) with robust standard errors.

For the NB rate model, the time period was the strongest individual predictor. CE lookups per visit were 43% higher in 2020-2021 compared to 2018-2019 (IRR = 1.43, 95% CI, 1.42-1.44, *P* < .001), after controlling for other variables. Role was also significant: residents used CE 23% more per visit than attending physicians (IRR = 1.23), while APCs used it slightly more (IRR = 1.02). Experience demonstrated a negative association with CE use: each additional year of experience resulted in a 0.6% decrease in CE usage per appointment (IRR = 0.994, 95% CI, 0.994-0.995, *P* < .001). Although the annual effect appears modest, the cumulative impact is substantial. For example, a clinician with 20 more years of experience than a peer would be predicted to have a CE lookup rate approximately 12% lower (0.994^20^ ≈ 0.88). Given the sample’s baseline intensity of approximately 0.45 lookups per appointment, this equates to about 0.05 fewer lookups per appointment, representing a meaningful difference when aggregated over a full clinic day with multiple patients. Gender was not a statistically significant predictor (IRR ≈ 0.99, *P* = .059). Figure 1 displays the adjusted incidence rate ratios (IRRs) and 95% confidence intervals for predictors of Care Everywhere lookups per appointment, based on the negative binomial model. A sensitivity analysis using generalized estimating equations (GEE) to account for repeated clinician-day observations yielded similar effect estimates; full GEE results are provided in Supplementary File S1. Full model coefficients, including IRR point estimates, 95% confidence intervals, and *P* value for all predictors, are provided in Table S3.

In clinician fixed-effects robustness models, the Poisson FE with a log-appointments offset estimated IRR(post) = 1.409 (95% CI, 1.38-1.43), indicating a 40.9% increase in CE lookups per appointment within clinicians in 2020-2021 compared to 2018-2019. These within-clinician estimates support the primary findings.

**Figure 2.**
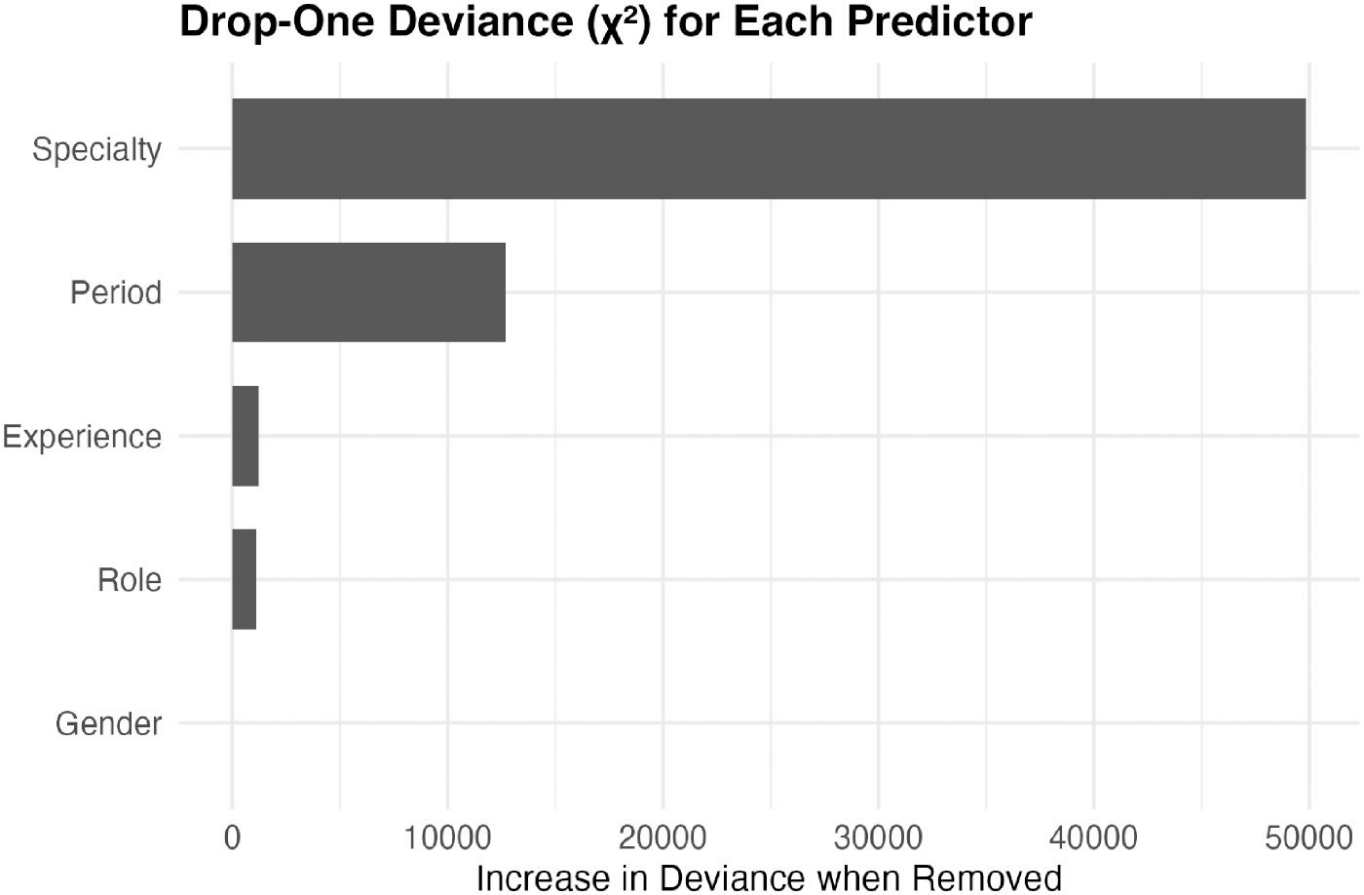
Drop-one deviance (Δχ^2^) for each predictor.

Although the study period exhibited the largest effect size, specialty accounted for the greatest overall model variance. To assess the relative importance of each predictor, a drop-one deviance test was carried out. Each variable was removed one by one, and the increase in deviance was measured. The findings indicated that specialty represented the largest portion of model deviance (Δχ^2^ ≈ 49 833), followed by period as the next most significant predictor (Δχ^2^ 12 669), and then experience and role. Gender made a minimal contribution to model fit (Δχ^2^ ≈ 4, *P* = .16). These results illustrated that the time period significantly impacted CE use behavior, while differences at the specialty level explained the most variation among clinicians in daily CE usage rates. The result of the drop-one deviance test for each predictor is shown in Figure 2.

## Discussion

Despite significant policy and technical advancements in facilitating interoperable data exchange, as EHR vendors and care delivery organizations adopting standards like FHIR-based APIs^30^ and planning to participate in TEFCA,^31^ actual interoperability use likely varies significantly based on clinician characteristics. Our results identified significant variation in clinician use with interoperability tools, specifically Care Everywhere (CE), based on time period, specialty, clinical role, and years of experience. CE use rose by 43% during the COVID-19 period (2020-2021) compared to the pre-pandemic period (2018-2019). The specialty of the clinicians was the strongest determinant of usage variation, with Nephrology and Cardiology consistently using more with CE, while Dermatology and Pediatrics utilized it substantially less. This variation may arise not only from the complexity of the care but also from the nature of the relationship between the clinician and patient. Studies have found that patients have significantly different experiences depending on whether their specialist visits are part of an ongoing relationship or a one-time consultation, and relationships that only involve consultations are associated with lower use and satisfaction.^32^ Furthermore, some specialists, such as cardiologists, often act as the primary care source, managing chronic conditions and coordinating referrals across different settings.^33^ These care models, tailored to specific needs, may create more incentives to retrieve and use external patient records to coordinate patients’ complex care needs. This distinction could help explain the observed differences in CE use across specialties. Our robustness check with clinician fixed effects confirmed that the pre/post increase also occurs within the same clinician, supporting the primary GEE findings.

Gender was not a significant predictor. Residents were 23% more likely than attendings to use CE per visit, and CE usage declined slightly with increased experience. This indicates that trainees and mid-level clinicians engage with external patient data more frequently than attending physicians. This pattern may suggest that outside records are particularly useful for less-experienced clinicians, who may more often encounter patients with limited prior clinician–patient history in a given setting; however, the data do not capture care continuity. The higher CE use among residents may partly reflect differences in patient panels rather than clinician-level preferences alone, as residents may disproportionately encounter new or more complex patients for whom outside records are particularly valuable. Without patient-level data, clinician behavior cannot be fully disentangled from the characteristics of the patients they serve. Future research linking CE use to individual encounter attributes such as whether encounters were new versus established or consultative versus longitudinal would help clarify this distinction. The results imply that younger or more recently trained clinicians may be more comfortable using interoperability tools and that pandemic-induced shifting of clinical demands amplified this reliance. One possible explanation is that clinicians may rely more on outside records when they have less prior knowledge of a patient; however, we do not directly observe patient-clinician familiarity or whether encounters were consultative vs longitudinal in these data. Our results align with past evidence indicating that many clinicians still struggle with unclear economic incentives,^34^ clinicians’ resistance due to challenges with system design (eg, extra clicks, uncertain clinical value, and usability hassles),^20^ and the overall complexity of integrating interoperability tools into their existing workflows.^35^ Improving the ease of navigation and information retrieval and adoption of interoperability tools like Care Everywhere is crucial to strengthening continuity of care, reducing redundant testing, and supporting informed, timely decision-making. The importance of these tools becomes even clearer when a patient is seeing multiple clinicians or managing a chronic disease. Interoperability tools have the potential to strengthen continuity of care and reduce redundant testing; however, evaluating downstream clinical outcomes, costs, or population health impacts was beyond the scope of this study.

### Limitations

First, this study is limited by its retrospective design. The analysis shows existing patterns of use but does not explain the underlying causes without additional qualitative or experimental research. Second, the study was conducted at a single academic health system, which may limit the generalizability to institutions with different referral patterns, payer mixes, or interoperability infrastructures. Third, patient-level characteristics, such as age, comorbidity burden, or insurance status, encounter type (new vs established patient), or whether a patient receives all care at UCSF, were not included as predictors of clinician CE use. Some of the variation attributed to clinician characteristics may therefore partly reflect differences in the patient panels they serve. Additionally, this study does not presuppose that CE use is uniformly required for high-quality care; rather, it documents observed variation in use and identifies clinician-level predictors of that variation. Future research should link clinician activity to patient attributes to determine whether specific patient populations face “missed” opportunities when relevant outside information is available but not accessed. This study also cannot directly quantify the volume or completeness of outside records available to each clinician through Care Everywhere on any given day, as this is determined by the patient’s care history and external care partner participation. A clinician’s decision not to look up records may therefore sometimes reflect an absence of available data rather than a behavioral preference. Future studies should, where possible, link usage metrics to data availability indicators. Additional work is also needed to understand why some specialties engage with CE more than others. It is important to assess the specific impact of COVID-19 on clinical information-seeking behaviors and the system-level or behavioral factors (eg, clinic size, patient complexity, and burnout) that influence CE use but were not captured in this dataset. Future research should also examine how the consult versus ongoing nature of the specialist-patient relationship and the role of specialties as primary-care clinicians affect clinician use of outside records.

## Conclusion

This study advances our understanding of clinician use of interoperability tools by highlighting significant variation across specialties, clinical roles, experience levels, and time periods. The increase in Care Everywhere usage during the COVID-19 pandemic demonstrates how contextual factors influence information-seeking behavior. Specialty is the strongest predictor of usage, with clinicians who manage more complex or chronic conditions on an ongoing basis showing higher use of interoperability tools, indicating that clinicians managing “whole person care” not only access external records more consistently, but also perform multiple lookups within single encounters. These findings indicate that improving interoperability requires not only a robust technical infrastructure but also targeted strategies to address the diverse needs and behaviors of clinicians. To maximize the clinical and public health benefits of interoperable EHR systems, it’s important to prioritize ease of navigation and information retrieval improvements, integrate interoperability competencies into clinical training, and maintain supportive policy incentives. Future research is needed to identify the underlying drivers of usage differences and to optimize adoption across healthcare settings.

## Supplementary Material

ooag116_Supplementary_Data

## Data Availability

The data used in this study were obtained from the UCSF electronic health record system under a data use agreement with the American Medical Association (AMA). Due to data privacy and institutional restrictions, the dataset cannot be shared publicly or deposited in a public repository such as DRYAD. Deidentified analytic outputs or code used in this analysis are available from the corresponding author upon reasonable request, subject to UCSF data governance policies.
