# Supplementary material for: Patterns and predictors of clinician use of interoperability tools
Source: JAMIA Open. 2026 Jun 11;9(4):ooag116. doi: 10.1093/jamiaopen/ooag116 (PMC13348719; doi:10.1093/jamiaopen/ooag116)
Supplement: ooag116_Supplementary_Data [file ooag116_supplementary_data.docx]

**Supplementary MATERIAL**

**Supplementary data are available at JAMIA online**

| **Day of Week** | **N-Active Days** | **Total Scheduled Appts** | **Avg Appt per Active Days** |
| --- | --- | --- | --- |
| Monday | 28863 | 250199 | 8.6 |
| Tuesday | 34116 | 290285 | 8.5 |
| Wednesday | 32602 | 271541 | 8.3 |
| Thursday | 31672 | 263161 | 8.3 |
| Friday | 27984 | 226032 | 8.0 |
| Saturday | 396 | 3039 | 7.6 |
| Sunday | 407 | 1509 | 3.7 |

Table S 1 Appointment by Weekday Distribution

| **Day Specialty** | **Number of Clinicians** |
| --- | --- |
| Internal Medicine | 368 |
| Neurology | 290 |
| OB/GYN | 181 |
| Pediatrics | 177 |
| Cardiology | 114 |
| Dermatology | 112 |
| Heme/Onc | 90 |
| Otolaryngology | 49 |
| Nephrology | 43 |
| General Surgery | 42 |
| Family Medicine | 29 |
| Internal Medicine | 368 |

Table S 2 Clinician Specialty Distribution

| **Predictor** | **Comparison / Level** | **IRR** | **95% CI** | **p-value** |
| --- | --- | --- | --- | --- |
| **Time Period (ref: 2018–19)** | | | | |
| Period | 2020–21 vs. 2018–19 | 1.430 | 1.420–1.440 | < 0.001 |
| **Clinical Role (ref: Attending Physician)** | | | | |
| Role | Advanced Practice Clinician vs. Attending Physician | 1.020 | 1.010–1.030 | < 0.001 |
| Role | Resident vs. Attending Physician | 1.230 | 1.220–1.250 | < 0.001 |
| **Years of Experience (continuous)** | | | | |
| Experience | Per additional year | 0.994 | 0.994–0.995 | < 0.001 |
| **Gender (ref: Female)** | | | | |
| Gender | Male vs. Female | 0.993 | 0.986–1.000 | 0.059 |
| **Specialty (ref: Cardiology)** | | | | |
|  | Nephrology vs. Cardiology | 0.970 | 0.951–0.989 | < 0.01 |
|  | Heme/Onc vs. Cardiology | 0.914 | 0.901–0.928 | < 0.001 |
|  | Internal Medicine vs. Cardiology | 0.871 | 0.861–0.882 | < 0.001 |
|  | Family Medicine vs. Cardiology | 0.846 | 0.831–0.861 | < 0.001 |
|  | Neurology vs. Cardiology | 0.778 | 0.768–0.788 | < 0.001 |
|  | OB/GYN vs. Cardiology | 0.711 | 0.701–0.720 | < 0.001 |
|  | General Surgery vs. Cardiology | 0.696 | 0.677–0.715 | < 0.001 |
|  | Otolaryngology vs. Cardiology | 0.580 | 0.571–0.590 | < 0.001 |
|  | Pediatrics vs. Cardiology | 0.380 | 0.373–0.387 | < 0.001 |
|  | Dermatology vs. Cardiology | 0.238 | 0.234–0.242 | < 0.001 |

Table S 3 Adjusted Incidence Rate Ratios (IRRs) from the Negative Binomial Rate Model: Predictors of Care Everywhere (CE) Lookups per Appointment

*IRR = Incidence Rate Ratio. CI = Confidence Interval. APC = Advanced Practice Clinician. Ref = reference category. p-values are one-sided; values ≥ 0.05 are not statistically significant.*

*Model: Negative binomial rate model with log(appointments per day) as offset. Reference categories: Female (gender); Attending Physician (role); Cardiology (specialty); 2018–19 (period). All IRR values and 95% CIs are exact estimates from the R model output (glm.nb, MASS package).*

*Note: Clinicians with unknown gender (n very small) were excluded from this table as this category was not reported in the main manuscript due to negligible group size and an uninformative confidence interval.*

**Generalized Estimating Equations (GEE) Analysis**

Generalized Estimating Equations (GEE) with a Poisson distribution were applied to address the correlation of repeated observations among clinicians. A working correlation structure was specified to adjust standard errors while estimating population-averaged effects. Exchangeable and independent correlation structures were compared. In the exchangeable correlation structure, it is assumed that there is a constant correlation among all days within a clinician, while independence assumes no correlation but uses robust standard errors to account for clustering. Model fit was assessed using quasi-likelihood information criteria (QIC), and the independence structure demonstrated a better fit (QIC = –462,443) than the exchangeable structure (QIC = –440,020). Thus, results from the independence-working-correlation GEE model were reported.

Key GEE findings confirmed the results of the negative binomial GLM. The time period was significantly associated with CE usage, showing a 42% increase in lookups per appointment during 2020–2021 compared to 2018–2019 (IRR = 1.42, p < 0.001). Specialty differences reflected those observed in the GLM results: dermatology, pediatrics, and OB/GYN had significantly fewer CE lookups per visit than cardiology, while specialties such as hematology/oncology and nephrology did not differ significantly from cardiology. Gender was not a significant predictor (male vs. female IRR ≈ 0.99), and role comparisons showed no statistically significant differences between residents or attending physicians and advanced practice providers. Years of experience indicated a marginal trend toward decreased CE use (approximately 0.4% decrease per year, p = 0.058).
